# Retention of the Gastric Contents

**Published:** 1913-11

**Authors:** Charles D. Aaron

**Affiliations:** Professor of Gastro-Enterology and Adjunct Professor of Dietetics in the Detroit College of Medicine; Consulting Gastro-Enterologist to Harper Hospital. Detroit


### BUFFALO MEDICAL JOURNAL

VOLUME 69 | NOVEMBER, 1913 | No. 4

### ORIGINAL ARTICLES

*The right is reserved to decline papers not dealing with practical medical and surgical subjects and such as might offend or fail to interest readers. Contributors are solely responsible for opinions, methods of expression and revision of proof.*

### Retention of the Gastric Contents

BY CHARLES D. AARON, Sc. D., M. D.

Professor of Gastro-Enterology and Adjunct Professor of Dietetics in the Detroit College of Medicine; Consulting Gastro-Enterologist to Harper Hospital, Detroit.

Presented to the Dominion Medical Association, London, Ontario, June 24-27, 1913.

IN discussing the subject of the retention of the stomach contents, normal gastric motility must be thoroughly understood. Gastric tonus is a state of more or less shortening of the circular muscle of the stomach.* Peristaltic waves are independent of intragastric pressure. The peristalsis of the stomach consists of a series of continuous waves following each other in rhythmic succession. Tone is necessary for normal gastric peristalsis. The function of the stomach is that of a reservoir, the contents of which it must deliver to the intestine in a definite time. In the interval the food undergoes certain changes which we term digestion. The food and secretions are churned up together and prepared before being emptied into the duodenum. The stomach does not empty itself by gravity drainage. The cardiac and pyloric portions of the stomach are divided by the angular notch, *incisura angularis*. It is the pyloric portion that has to do with propelling the food onward by deep peristaltic waves; the cardiac portion contracts and thus presses the food on toward the pylorus. The strong peristalsis in the pyloric half of the stomach churns the food to the consistency of cream. As long as food remains in the stomach this peristaltic action continues, but it stops at the pyloric orifice; it does not pass on into the duodenum.

Hydrochloric acid in the stomach relaxes the pylorus. A delay in the acid reaction in the pyloric region causes retention of food in the stomach in spite of strong peristalsis. When the acid

* W. B. Cannon, The Mechanical Factors of Digestion, 1911

chyme is discharged into the duodenum, the pylorus closes, for acid in the duodenum has an effect upon the pylorus directly opposite to that of acid in the stomach. The closure of the pylorus continues until all the acid in the duodenum has been neutralized, when the acid in the stomach again relaxes the pylorus and the chyme is injected into the duodenum. This alternation of movement, the opening and closing of the pylorus, continues until the stomach is completely emptied.

Retention of the stomach contents is not a disease in itself, but a symptom complex. Different names have been given it, such as dilatation of the stomach, ectasia ventriculi, gastrectasis, ischochymia, and motor insufficiency of the second degree. The cause of the condition is usually an obstruction of the pylorus; and since the pyloric stenosis eventually produces a complete pathologic picture, it has become customary to speak of gastric retention as a separate affection.

In retention due to stenosis the first effect upon the musculature of the stomach is hypertrophy; but as the pyloric obstruction becomes less and less permeable, muscular weakness and gastric dilatation result.

If the pylorus is functionally inefficient owing to a constricted lumen, motor disturbance must follow. The fundus may be compared to the auricle and the pylorus to the ventricle of the heart. In mitral regurgitation there is at first a compensating hypertrophy of the left ventricle; but as soon as this compensation is broken, dilatation occurs. This same process of hypertrophy and dilatation of the stomach musculature occurs in stenosis of the pylorus.

At one time the opinion prevailed among clinicians that abnormality in the size or position of the stomach was largely responsible for motor disturbances. It has been found, however, that greatly dilated and ptotic stomachs do not of necessity interfere with gastric motility. They are not pathologic per se.

The absolute size of a stomach is of no significance; a large and apparently dilated stomach may be functionally efficient. Ewald has given the name "megalogastria" to the normally large stomach. The material point in the diagnosis of gastric retention is not size, but stagnation of contents. A stomach in which the food contents are stagnant because they cannot be evacuated is dilated in the clinical sense.

A stomach in health should empty itself of a large meal in seven hours. The term "motor insufficiency of the first or second degree" has been given to disturbances in the motility of the stomach. In motor insufficiency of the first degree the evacuation of the stomach, though complete, is retarded. In motor insufficiency of the second degree the stomach has entirely lost the ability to expel its contents; that is, food residues remain in the stomach permanently, inducing stagnation; and as a consequence of this chronic condition of gastric insufficiency, dilatation of the stomach ensues.

Motor insufficiency of the first degree is contingent upon a primary relaxation of the muscular wall of the stomach, or a loss of gastric tonus. This condition, which is often found, is known clinically as gastric atony or myasthenia. In gastric atony the muscles may be so greatly distended by large quantities of food as to constitute a condition of transient dilatation of the stomach, but this condition must be differentiated from established or permanent dilatation. Should a person with a normal musculature drink a sufficient quantity of water, the lower border of the stomach would descend to the level of the umbilicus, as shown by gastric dullness or the X-ray; but an atonic stomach may be so distended by fluids that the lower border falls below this point. Splashing sounds elicited when the stomach should be empty, help to confirm the diagnosis of atony. The stomach in a condition of atony contains food remnants seven hours after the ingestion of a full meal; it, however, empties itself completely during the night after a full meal. This is the all-important point in arriving at a positive clinical diagnosis of atony.

In motor insufficiency of the second degree there is an obstruction to the pyloric outlet. The musculature is hypertonic rather than atonic. The gastric walls are hypertrophied from the peristaltic movements of the stomach in its persistent efforts to empty itself. Careful clinical and anatomic examinations have shown us that stenosis of the pylorus is the cause of motor insufficiency of the second degree. The lumen of the pylorus may be narrowed from the inside or from the outside; it may be cicatrized and contracted from the healing of gastric ulcers, or there may be cicatricial tissue as a result of healed perforations from biliary calculi. Spastic stenosis of the pylorus is by no means a rare condition; it is caused by the irritating effect of the ingesta upon erosion or fissure of the pylorus or by an abnormally high degree of gastric acidity. This closure of the pylorus is at first periodic (pylorospasm), but when the attacks become more frequent there results a permanent stenosis. Hypertrophy of the pylorus may result from chronic gastritis, angulation from gastroptosis, perigastric adhesions, epigastric hernia, or repeated injuries in this portion of the stomach. In hypertrophic changes in the pylorus, the process is slowly progressive. These cases pass from mechanical motor insufficiency (atony) to motor insufficiency of the second degree (dilatation).

Syphilis may become an etiologic factor in chronic hypertrophy of the pylorus. Internal stenosis resulting from malignant tumors is by no means rare. Polypi and myomata are occasionally met with. Adhesions of the stomach to neighboring organs or to abdominal tumors may cause pyloric stenosis by compression or by bending the pylorus upon itself. Among the more remote causes a right movable kidney may be mentioned.

Though pyloric stenosis may be the starting point of gastric dilatation, this result does not follow so long as the stomach musculature is capable of overcoming the obstruction. Stenosis which develops slowly leads to hypertrophy of the musculature precisely as in valvular insufficiency. This compensation may render efficient service for a long time—under certain circumstances for life. But indiscretion in diet may throw too much work upon the hypertrophic musculature, or the stenosis may increase by cicatrization; in either case, retention of the gastric contents is apt to follow, with consequent dilatation of the stomach. We may say that dilatation of the stomach is a pyloric affection in the stage of disturbed muscular compensation.

It was not until Kussmaul introduced the use of the stomach tube that a complete revolution in the diagnosis and treatment of retention of the stomach contents was rendered possible. A precise diagnosis cannot be made without the aid of the stomach tube; but with this aid and intelligent, systematic examination of the stomach contents, the physician may make an early and accurate diagnosis and save the patient much suffering.

Next to ulcer, carcinoma of the pylorus is the most important and dangerous cause of occlusion of the pyloric lumen. The differentiation is often a very difficult one. A tumor may be so small and smooth as to entirely escape palpation, and there may be nothing but the motor disturbance to indicate stenosis.

A grave complication of ulcer is perigastritis. Adhesions may change the lumen of the pylorus by displacement and distortion. Cholecystitis may lead to the same result, as it may impede the motility of the pylorus by adhesions, forming the cobwebs of Morris. I recall a case of gastric retention where all the clinical symptoms pointed to a carcinomatous affection. Stagnation, with lactic acid, pus and blood in the gastric contents were present. At operation the stomach was dilated, but there was no tumor. Instead, there was found an infected gall-bladder, with gall-stones and adhesions. The gall-stones were removed, the bladder drained, and the patient made a complete recovery.

An exact anamnesis assists greatly in making out the actual cause of the affection. The various data of the entire course of the affection should be carefully recorded before a physical examination is made. An affection that dates back several years is probably not malignant, but it should always be remembered that an old ulcer may have become carcinomatous.

By far the most important symptom of gastric retention is vomiting, which is usually profuse. At first it does not occur often, but the intervals continue to grow shorter until at last large quantities, apparently larger than those ingested, are vomited every day. The vomitus will contain food remnants many days old, for food that is not readily digestible may remain in the stomach for weeks.

On standing in a sedimentation glass, the vomitus usually separates into three layers. The solid particles, being the heaviest, sink to the bottom; the fluid above is cloudy, and the top layer consists of more or less viscid mucus permeated by gas bubbles. This stratification in three layers is thoroughly characteristic of all forms of gastric retention, which are due to or associated with stenosis of the pylorus.

The vomiting of malignant stenosis is totally different, especially after the affection has reached an advanced stage. The vomitus is no longer dilute, but viscid—like a thick soup and permeated by mucus masses, everything being so closely intermixed that it is difficult to diffuse the mass with water. The odor is peculiarly mouldy, sometimes absolutely putrid like decomposed tissue. The food remnants are almost unchanged. Meat can be found days after being taken into the stomach, and even farinaceous food is undigested. The appearance of the gastric contents is so characteristic as to be almost sufficient of itself to determine the diagnosis. On the other hand, there may be demonstrable carcinoma when the gastric contents do not present this characteristic appearance.

Admixture of blood in the gastric contents is less frequent in benign stenosis. According to quantity and the time the blood has been stagnant in the stomach, its color varies from light yellow to dark brown. At times the admixture of blood can only be detected microscopically or chemically. Persistent occult hemorrhages occur in malignant stenosis, and they can be demonstrated both in the stomach contents and in the feces. Profuse hematemesis occurs much more frequently in benign stenosis with ulcer than in the malignant form.

The patient often suffers severe pains, which will not subside until the stomach has been emptied by vomiting or lavage. Or the sensation may be simply one of pressure, fullness and discomfort. The severe pains are probably the consequence of exaggerated distention from the gases of fermentation. As soon as the patients learn that evacuation of the stomach gives relief they purposely induce vomiting by putting a finger into the throat.

A very uncomfortable manifestation is the constant thirst. Since the water taken into the stomach is neither absorbed nor passed into the duodenum, the body becomes impoverished for fluid. That as much water as possible may be retained within the body (another instance of physiologic compensation), less than the normal proportion is eliminated by the kidneys. Patients are often unable to speak on account of the dryness of the tongue. There is usually constipation, which increases with the increase of stagnation. Insufficient absorption of water and the daily loss of water are responsible for this condition.

Nutrition suffers in all cases, whether the cause of the pyloric affection be malignant or not. Losses in weight of forty or fifty pounds are by no means rare. There are also characteristic peculiarities in the appearance of patients. If the nutritive disturbances are very considerable and there occurs an exacerbation of stagnation and fermentation in debilitated patients, the inanition may result in grave delirium, which will subside if it is possible to improve the digestion.

The examination of patients should include the entire body. Inspection of the abdomen may furnish valuable information. Peristaltic waves from left to right and gradually disappearing at the pylorus may occasionally be seen. This manifestation is most important and decisive of pyloric stenosis. The whole stomach may contract and its outline easily be seen through the abdominal wall. But this phenomenon is not always pronounced and may be absent; cold seems to elicit it most readily. By palpating the wall of the abdomen during this manifestation of "stomach stiffening," the curvature can be distinctly perceived.

The succussion sound has been erroneously looked upon as a pathognomonic sign of dilatation of the stomach. In nearly every individual who has ingested large quantities of fluids and whose abdominal walls are somewhat atonic, the succussion sound can be elicited; consequently, a symptom of this kind can only be utilized for diagnostic purposes with a certain measure of precaution. The succussion sound is suspicious if it occurs with an empty stomach in the morning or several hours after a meal, providing no large quantities of liquids have been ingested; but a diagnosis which depends exclusively upon this phenomenon is altogether unreliable.

A question of importance is whether the succussion sound occurs above the umbilicus or below. It is difficult to tell whether the sound proceeds from the stomach or from the intestines, and for this reason great caution is necessary. The succussion sound, present at a time when the stomach should be empty, presupposes a large atonic stomach and is suggestive of dilatation.

The size of the stomach can be easily determined by auscultatory percussion. Inflation of air or carbon dioxide for that purpose is frequently done. The X-ray after ingestion of bismuth is most reliable. When there is stenosis the shadow shows a distorted duodenal cap. After the size of the stomach has been determined, its capacity of distention can be learned by percussing the lower border during lavage after one pint of water has been introduced. The level to which the dullness sinks can in this way be clearly made out.

But nothing short of the stomach tube can furnish a correct diagnosis. The normal stomach should be empty six to seven hours after a test meal, and if the stomach is washed out at this time stagnation, if present, is easily discovered. Again, the patient is given with the evening meal, rice, raisins and prunes, and the contents of the stomach are removed the next morning before breakfast. If the stomach is found to be empty, there is no retention, no matter what size the organ has attained. The persistent presence of food remnants in a fasting stomach is decisive of retention.

Demonstration of lactic acid and the determination of the reaction for hydrochloric acid are of decided importance in determining whether a stenosis is benign or malignant. In nearly all cases of retention which have developed from a pyloric ulcer or its scar, there is hyperchlorhydria. In primary carcinoma there is almost always achlorhydria. Carcinoma must never be excluded, however, because hydrochloric acid secretion is normal or excessive, for when a carcinoma develops on the site of an old ulcer, hyperchlorhydria may persist for life. A decision can only be made after continued observation. The demonstration of occult blood in the feces is of great importance.

Sarcinæ and yeast cells are often seen microscopically. Sarcinæ thrive best in the presence of hydrochloric acid, and when found in the gastric contents are suggestive of benign stenosis.

Malignant stenosis is characterized by the presence of the lactic acid bacillus of Oppler-Boas, a sign which is almost pathognomonic. However, the demonstration of these bacilli alone should never be made the basis of a diagnosis, as deception is possible.

X-ray examination should never be neglected. In all doubtful cases the presence or absence of a tumor can rapidly be established by means of it, and if a tumor is found to be present the case can be referred to the surgeon at an earlier date than would have been possible with any other method of examination.

The most difficult problem the diagnostician faces is early proof of the benignness or malignity of the underlying affection. This is important because the therapy depends upon it. In case of malignity, nothing but its early recognition can save the patient. We should endeavor, therefore, to arrive at as early a decision as possible by resorting to all applicable methods. Unfortunately, our knowledge and methods of examination are not sufficiently advanced to enable us to make an early and reliable diagnosis with absolute certainty.

The difficulties are least in the presence of a primary pyloric carcinoma. The rapid increase of the stenosis, the accompanying severe mucous catarrh of the stomach, the presence of lactic acid, deficiency of hydrochloric acid, the presence of the Oppler-Boas bacillus, rapid loss of weight, increase of stagnation in spite of careful dieting and regular lavage, occult blood in the feces, and, above all, the demonstration of a pyloric tumor, should secure a prompt diagnosis. But even if no tumor can be felt, a negative diagnosis cannot be safely made without an X-ray examination. Explorative laparotomy does not always admit of a definite decision as to whether there is carcinoma or not.

A secondary carcinoma which has developed on the site of an old ulcer often presents insurmountable obstacles to an early diagnosis. Knowing as we do that an ulcer very frequently undergoes carcinomatous degeneration (Mayo), early operation is urgently to be advised.

The treatment of gastric retention is at first always internal; it would be a great mistake to refer every case to the surgeon. The most important point is the diet, which will vary according to the degree of acidity of the stomach contents and the chronicity of the affection. Meat is practically undigestible in cases of anacidity.

Since it is impossible to supply sufficient water for the body's wants by way of the mouth, it is advisable to give small saline enemas several times in the course of the day, or to introduce water through the rectum by the drop method.

Lavage of the stomach is the sovereign means of unloading the stomach, removing fermentation and decreasing the catarrhal condition. The irrigations should be carried out very thoroughly, for they frequently improve the appetite and stop vomiting.

Cohnheim's oil treatment is often attended with excellent results. In benign stenosis, surgical intervention should never be resorted to until the patient has been given the benefit of the oil cure. The latter will also favorably influence hypersecretion.

Wearing a well fitting stomach binder will give patients great comfort, as it will afford support to the enlarged stomach, facilitating its evacuation.

If in spite of rational treatment, including the dietary, irrigations, etc., retention cannot be influenced, if the urine does not increase in quantity, if there is loss of weight, and if no improvement is obtained either by the rest cure or by the oil treatment, then operation should be advised, even in cases of benign stenosis. Surgical intervention is absolutely indicated as soon as malignity is discovered, unless there be marked metastasis or the tumor has attained to such proportions as to frustrate every hope of success by radical operation. Unfortunately, the idea held out by some surgeons, that with the performance of laparotomy the whole situation is cleared up, is absolutely wrong, for carcinoma cannot with certainty be distinguished from an inflammatory tumor without the microscope. As a matter of course, early operation should always be advised in suspicious cases.

I have used Einhorn's pyloric dilator with remarkable success in one case of retention due to benign stenosis. The patient was a man forty-eight years of age. He had been sick nine years. For the last five years the great discomfort he experienced compelled him to wash out his stomach frequently. Pain, and sometimes vomiting, would come on about two hours after meals. At times he would vomit enormous quantities of food that he had taken days before. He had lost thirty pounds in weight; was tired quickly; had backache and was constipated. His stomach contents showed stagnation, with hyperchlorhydria. No Oppler-Boas bacilli could be found, but there was an abundance of sarcinæ. The feces contained no occult blood. The use of the pyloric dilator brought about an apparently complete recovery. The dilator was used only three times during twelve days. The patient improved so rapidly that it was unnecessary to do any more stretching of the pylorus. He regained his weight and strength. It is now eighteen months since the last stretching.

The pyloric dilator consists of a thin rubber tube 80 centimeters long, with a small metal end piece. Next to this metal piece and fastened to it and the tube is a tiny rubber balloon covered with silk gauze. The portion of the tube covered by the balloon is perforated in several places for the passage of air into the balloon, and at the upper end of the tube there is a stopcock and a graduated glass syringe, the latter for inflating the balloon. The balloon being emptied of air, the cock is closed. The end piece of the dilator is dipped into warm water and introduced into the patient's pharynx. The patient drinks some water and the dilator moves into the stomach, where it is left undisturbed over night. In the morning, the balloon now being in the duodenum, the stretching is performed. The balloon is first inflated, and as the tube is drawn upward and forward there is a sensation as if the end of the dilator were held tight by something that drags along with it. The balloon is then slightly deflated by drawing on the piston of the syringe. This is done repeatedly until the end of the dilator passes upward through the pylorus.

32 West Adams Avenue.

---

Influence of X-rays on Germination. Drevon, *Arch. d'Elec. Med.,* July 10, 1913, page 45, finds a stimulant action on sprouting grains, with mild irradiation, especially combined with warmth. In the discussion, the question was raised whether strong rays would not have a deterrent effect in accordance with the general principle observed for other therapeutic means that a difference of dose, or strength causes opposite effects. The question was not definitely answered. (Note—When we consider the well known application of this principle to various drugs, mechanic methods, heat, and other forms of radiant energy, it is only fair to acknowledge that there is something to the doctrine of similia similibus).

---

Treatment of Amoebic Dysentery and Liver Abscess, Dessy and Marotta, *La Semana Medica,* April 3, 1913. Dessy and Marotta report a case treated according to the method recommended by Rogers of Calcutta. This treatment is only effective in the amoebic form of dysentery. 2-6 centigrammes of emetin are administered by subcutaneous injection daily. Their patient had 32 bowel movements a day. Amoebæ in large numbers were found. The effect of the emetin was immediate; the stools were greatly reduced in number, less liquid, less mucus, and on microscopic examination, numbers of dead amoebæ were found. Four injections in all were given, when the patient had apparently returned to normal.

